# Cryptogenic Organizing Pneumonia with a Rare Radiographic Presentation of a Diffuse Micronodular Pattern Mimicking Miliary Lung Infiltration: A Case Report and Review of the Literature

**DOI:** 10.1155/2020/2094625

**Published:** 2020-01-03

**Authors:** Jakrin Kewcharoen, Kittika Poonsombudlert, Sakda Sathirareuangchai, Wichit Sae-Ow, Hanh La, Narin Sriratanaviriyakul

**Affiliations:** ^1^University of Hawaii Internal Medicine Residency Program, Honolulu, HI 96813, USA; ^2^University of Hawaii Pathology Residency Program, Honolulu, HI 96813, USA; ^3^The Queen's Medical Center, Division of Pathology, Honolulu, HI 96813, USA; ^4^The Queen's Medical Center, Division of Internal Medicine, Honolulu, HI 96813, USA; ^5^The Queen's Medical Center, Division of Pulmonary & Critical Care Medicine, Honolulu, HI 96813, USA; ^6^Department of Medicine, University of Hawaii at Manoa, John A. Burns School of Medicine, Honolulu, HI 96813, USA

## Abstract

We reported a case of cryptogenic organizing pneumonia (COP) presenting with an unusual diffuse micronodular pattern (DMP) mimicking miliary lung infiltration. The patient is a 66-year-old man with a past medical history of diabetes mellitus type 2 and hyperlipidemia who presented with progressive dyspnea associated with significant weight loss and night sweats for 2 weeks. Upon admission, the patient's clinical condition rapidly progressed to respiratory failure requiring mechanical ventilation. Initial Chest X-ray (CXR) showed diffuse reticulonodular infiltration mimicking miliary pattern. Chest computed tomography (CT) showed diffuse centrilobular micronodular infiltrations with features of a tree-in-bud pattern consistent with the CXR findings. He was then started on empiric antibiotics for community-acquired pneumonia and underwent a diagnostic bronchoscopy with alveolar lavage and transbronchial biopsies, which yielded negative cultures and unrevealing pathology. Tissue from CT-guided lung biopsy performed later on was also inconclusive. Due to the lack of clinical improvement, he eventually underwent surgical lung biopsy. The pathology result showed organizing pneumonia (OP) pattern with heavy lymphoplasmacytic infiltrates and numerous multinucleated giant cells. His final culture results, microbiological data and serology workup for autoimmune disease were all unremarkable. The patient was diagnosed with COP and was started on systemic corticosteroids. He displayed dramatic clinical improvement and was successfully liberated from the ventilator. Subsequent chest imaging showed resolution of the reticulonodular infiltrations. Early diagnosis for OP and ability to distinguish OP from infectious pneumonitides are critical as the majority of patients with OP respond promptly to corticosteroids. Common findings of radiographic pattern for OP are patchy air space consolidation or ground-glass opacity, yet DMP is another rare radiographic pattern that must be recognized, especially in COP. In summary, this case illustrates a rare radiographic presentation of COP. With early recognition and prompt diagnosis, proper treatment can significantly prevent morbidity and reduce mortality.

## 1. Introduction

Organizing pneumonia (OP), formerly known as bronchiolitis obliterans organizing pneumonia (BOOP), is a specific type of diffuse interstitial lung disease that affects the small airways, primarily within the alveolar wall [1, 2]. Usual radiologic findings of OP consist of ground-glass opacification and/or consolidation distributed along the bronchovascular bundles with peripherally or subpleural predominant on a chest X-ray (CXR) [2, 3]. Computed tomography (CT) of the chest usually shows bilateral migratory patchy alveolar opacities that resolves spontaneously [1, 3]. Other uncommon radiographic patterns that could be found in OP include focal pneumonia, perilobular consolidations, curved bands of consolidation, single or multiple nodules, and diffuse micronodular pattern (DMP) [1, 4, 5]. To make a diagnosis of OP is challenging even with the usual radiologic findings as patients often present with non-specific respiratory symptoms which are common in infectious pneumonitides, including shortness of breath, cough and fever. Because of this, OP is commonly misdiagnosed as infectious etiology and initial treatments would often include anti-microbial agents. Eventually, patients would undergo invasive procedure for tissue diagnosis, which is characterized by buds of granulation tissue progressing from fibrin exudates to collagen-containing fibroblasts and myofibroblast proliferation intermixed with loose connective tissue in the distal air spaces [1, 6].

Organizing pneumonia is known to be associated with various conditions including connective tissue diseases, infectious processes, side effects from medication, acid reflux disorder, reaction from organ transplant or near-by malignant process [1, 6–8]. When there are no identifiable causes of OP, it is termed cryptogenic organizing pneumonia (COP). Apart from the typical radiographic findings, DMP mimicking miliary lung infiltration as a radiographic presentation in COP is extremely rare. This particular infiltration pattern in OP/COP is termed micronodular organizing pneumonia (MNOP) with only a few cases exist in the literature. We reported a case of COP presenting with DMP. We also performed a review of literature of COP patients who have a radiographic presentation of DMP.

## 2. Case Presentation

**Figure 1:**
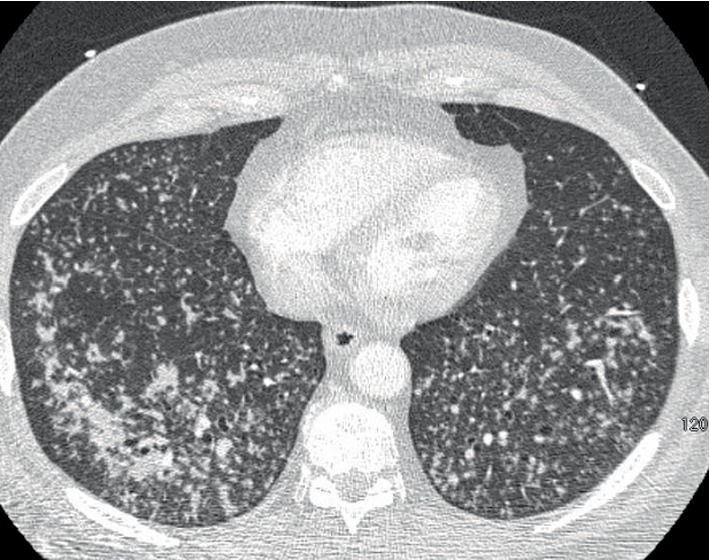
Computer tomography of the chest with contrast demonstrating bilateral diffuse centrilobular micronodular infiltrations with features of the tree-in-bud pattern.

**Figure 2:**
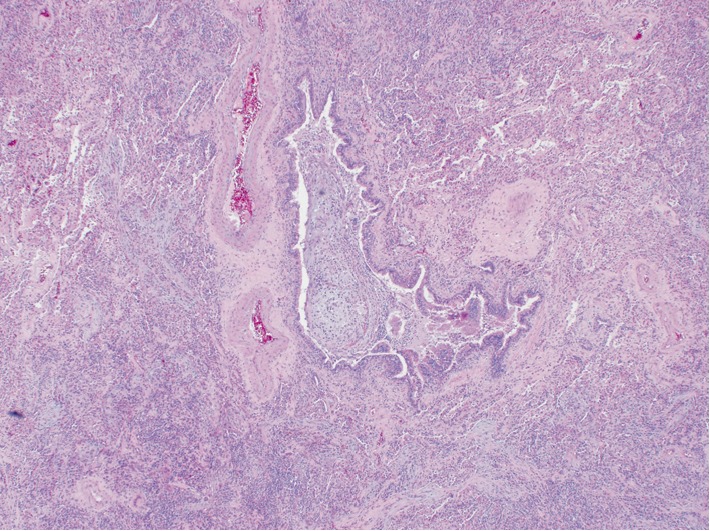
Pathology section from video-assisted thoracic surgery demonstrating fibroblast proliferation in terminal bronchioles extending into surrounding alveoli.

**Figure 3:**
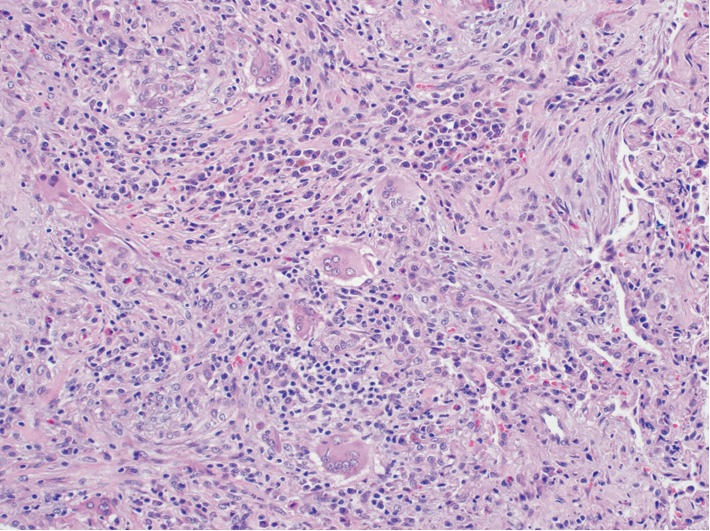
Pathology section from video-assisted thoracic surgery demonstrating heavy lymphoplasmacytic infiltrate and numerous multinucleated giant cells.

The patient was a 66-year-old man with a past medical history of well-controlled diabetes mellitus and hyperlipidemia. The patient had a 10-pack-year smoking history which he reportedly quit 50 years ago. He presented with progressive shortness of breath with dry cough, weight loss and night sweats for two weeks prior to admission. His physical exam at the time was pertinent for severe tachypnea, use of accessory respiratory muscles and bilateral lung crackles His initial CXR showed diffuse reticulonodular infiltration mimicking miliary pattern, and chest CT showed bilateral diffuse centrilobular micronodular infiltrations with features of the tree-in-bud pattern (Figure 1). He was initially treated empirically as severe community-acquired pneumonia with intravenous (IV) Ceftriaxone and Azithromycin but his symptoms did not improve appropriately. The patient's clinical condition rapidly progressed to respiratory failure requiring intubation and mechanical ventilation. Diagnostic flexible bronchoscopy with transbronchial biopsy and bronchoalveolar lavage was performed. Results yielded benign bronchial tissue without evidence of infection, inflammation or malignancy. Connective tissue diseases screening including rheumatoid factor, ANCA screen, Anti-nuclear Ab, Anti-Ro, Anti-La, Anti-CCP were unremarkable. All other infectious workup including blood and sputum culture, sputum acid-fast bacilli (AFB) stain, urine Legionella and Histoplasma antigen, serum Aspergillus and respiratory syncytial virus (RSV) antigen, antibody to *Mycoplasma, Bartonella henselae, Brucella, Coxiella burnetii* and *Coccidioides* was also unremarkable. After the initial treatment, the patient continued to display persistent lung crackles, thus a CT-guided lung biopsy was performed, which showed normal lung tissue with mild fibrosis and granulation and evidence of chronic inflammation without atypical cells or granuloma. Eventually, the patient underwent video-assisted-thoracic surgery (VATs) for tissue biopsy. Microscopic examination showed centrilobular nodular lesion of fibroblast proliferation in terminal bronchioles extending into surrounding alveoli, consistent with organizing pneumonia pattern (Figure 2). The lesion was also associated with heavy lymphoplasmacytic infiltrate and numerous multinucleated giant cells (Figure 3). Abundant fibrin and hyaline membrane were visible at the periphery of centrilobular nodules, consistent with diffuse alveolar damage pattern. There was no definite granulomatous inflammation, foreign body material, or geographic necrosis. Special stains for AFB and fungal organism were negative. After excluding all possible causes of pneumonitides, the diagnosis of COP was made. The patient was started on a systemic steroid course, IV methylprednisone 1 gram per day. He displayed remarkable improvement in his breathing and oxygen requirement and was finally liberated from the ventilator on the third day of the IV methylprednisone. He was then switched to oral prednisone 95 milligrams per day. His condition continued to improve steadily over the hospitalization and was later discharged with a decreased dose of oral prednisone, 65 milligrams per day, which was continued to be tapered outpatient. His follow-up CT subsequently showed resolution of the micronodular infiltration. He is currently doing well clinically at 2 years post-hospitalization.

## 3. Discussion

Organizing pneumonia is a well-known disease which has its own clinical and radiographic findings. It is mainly found in patients age between 50 and 60 years old, equally between male and female, with common symptoms being cough, shortness of breath and fever [7, 9, 10]. Interestingly, the prevalence was found more commonly in non or former smokers rather than active smokers [6]. Nevertheless, it is much less common than infectious lung diseases, thus patients are often mistreated with antimicrobial agents and deteriorate rapidly before the diagnosis of OP could be made [11, 12]. Common radiographic presentation includes patchy air space consolidation areas with a migratory course and ground-glass opacities predominantly in the lung periphery [9, 13]. Other less common patterns had been reported including perilobular or focal consolidations, curved bands of consolidation, and DMP [1, 4, 5].

In general, the prognosis of COP is good as most forms of COP will respond very well to steroid regardless of the infiltrative pattern on the imaging, if given promptly. Nevertheless, many patients end up being on a long-term steroid therapy due to a high rate of relapse [14] and up to 73% of the patients with COP would have residual disease seen on follow-up CT. In such cases, the lesions generally resemble a fibrotic nonspecific interstitial pneumonia pattern that does not resolve even with complete clinical recovery [15].

Organizing pneumonia with DMP mimicking miliary lung infiltration was also termed “micronodular organizing pneumonia”. This particular pattern, both cryptogenic and secondary, was reported to be around 10–24% among all OP in the current literature [7, 13, 16]. The pattern of pulmonary infiltration in OP was believed to be related to patient's underlying immune status and whether there was an associated cause or idiopathic [7]. However, there was no clear association found in several case series, especially for MNOP [13, 16]. In our article and review, we focused mainly on COP with DMP mimicking miliary lung infiltration.

In our case, the microscopic findings were consistent with OP pattern. Geographic necrosis, which is found in granulomatosis with polyangiitis (Wegener granulomatosis), is not identified. Due to the presence of heavy lymphoplasmacytic infiltrate, IgG and IgG4 stains were performed to rule out IgG4-related interstitial lung disease. It was shown that IgG4 was presented in only minority of plasma cells, excluding the diagnosis of IgG4-related interstitial lung disease. All possible infectious etiologies have been worked up and showed negative result. Overall, given his dramatic response to steroids and all etiologies have been excluded, COP is the most likely diagnosis in this case.

We conducted a literature review of COP with DMP. We searched PubMed and EMBASE database from inception to May 2019 with search term including “cryptogenic organizing pneumonia”, “micronodules”, “micronodular organizing pneumonia”. We found a total of 6 cases of COP who presented with DMP [7, 15, 17–20]. We excluded one case as a full article was not available [19]. Thus, we include a total of 6 cases in our literature review, including our case. We found that the patients were mostly men (66%) with age ranged from 19 to 66 years old. History of active smoker was documented in only 1 case, while the rest were non or former smoker. Cough and dyspnea were presented in all patients. Fever was common (66%) while sputum production was not reported in any patient. Duration of symptoms ranged from 1 day to 2 weeks. Regarding radiographic findings, centrilobular distribution were the most common findings (83%), with only one case presented with nonspecific DMP. One patient had acute myeloid leukemia while every other patient was immunocompetent. We could not find any association between COP presenting with DMP with the immune competency of the patients. We believed that our patient is not immunocompromised. There was no significant comorbidity aside for well-controlled diabetes. Thus, the evidence suggests that MNOP could also occur in immunocompetent patients. Every patient eventually underwent invasive diagnostic procedure.  Table 1 summarized the characteristics of the 5 reported cases of COP presenting with DMP.

## 4. Conclusion

Cryptogenic organizing pneumonia can present with many radiographic patterns. Diffuse micronodular pattern is considered a rare presentation in COP which exists only in the literature. Despite DMP not being commonly found in patients with COP, lack of response to anti-microbial or other initial treatment should prompt physicians to consider other possible diagnoses including OP/COP. With appropriate treatment and timing, patients with COP usually recover uneventfully.

## Figures and Tables

**Table 1:** Summary of previous case reports of cryptogenic organizing pneumonia with imaging presentation of diffuse micronodular pattern.

First author, year	Gender, Age (years)	Presenting symptoms	Duration	Smoking history	Comorbidity	Pulmonary exam findings	Hypoxemia/hypoxia at presentation	Micronodule pattern	Initial treatment	Diagnostic procedure
Bots, 2012	Male, 19	Cough, dyspnea	N/A	N/A	N/A	N/A	N/A	Centrilobular	Moxifloxacin	OLB
Ko, 2009	Female, 49	Cough, dyspnea, fever	1 day	None	AML	Tachypnea, diffuse crackles	No	Nonspecific	Broad-spectrum antibiotic	VATS
Langen, 2007	Female, 28	Cough, dyspnea, fever	1 week	None	None	N/A	Yes	Centrilobular	Quadruple therapy for TB	OLB
Lebargy, 2017	Male, 38	Cough, dyspnea, fever	8 days	Current	None	WNL except tachypnea	Yes	Centrilobular	N/A	VATS
Turner, 2003	Male, 47	Cough, dyspnea, fever	8 days	None	None	Tachypnea, diffuse crackles	Yes	Centrilobular	Broad-spectrum antibiotic with prednisone	TBB
Our case	Male, 66	Cough, dyspnea	2 weeks	Former	Diabetes mellitus, hyperlipidemia	Tachypnea, diffuse crackles	Yes	Centrilobular	Ceftriaxone and azithromycin	VATS

AML: Acute myeloid leukemia, N/A: not applicable, OLB: open lung biopsy, TB: tuberculosis, TBB: transbronchial biopsy, VATS: video-assisted thoracic surgery, WNL: within normal limit.
